# Cross-Taxa Similarities in Affect-Induced Changes of Vocal Behavior and Voice in Arboreal Monkeys

**DOI:** 10.1371/journal.pone.0045106

**Published:** 2012-09-12

**Authors:** Alban Lemasson, Kevin Remeuf, Arnaud Rossard, Elke Zimmermann

**Affiliations:** 1 Université de Rennes 1, Ethologie animale et humaine, UMR 6552 – CNRS, Station Biologique de Paimpont, Paimpont, France; 2 Institut Universitaire de France, Paris, France; 3 Institute of Zoology, University of Veterinary Medicine Hannover, Hannover, Germany; University of New England, Australia

## Abstract

Measuring the affective state of an individual across species with comparable non-invasive methods is a current challenge in animal communication research. This study aims to explore to which extent affect intensity is conveyed in the vocal behaviours of three nonhuman primate species (Campbell's monkeys, De Brazza's monkeys, red-capped mangabeys), which vary in body size, ecological niche and social system. Similarly in the three species, we experimentally induced a change in captive social groups' affect by locking all group members together in their outside enclosure. The two experimental conditions which varied in affect intensity consisted in imposing a pre-reunion 90 mn-separation by splitting up the respective group into two subgroups (High affect condition) or not (Low affect condition). We measured call rates as well as voice features at the time of reunion in both conditions. The three studied species reacted in a very similar way. Across species, call rates changed significantly between the behaviourally defined states. Furthermore, contact call duration and, to some extent, voice pitch increased. Our results suggest, for the first time in arboreal Old World monkeys, that affect intensity is conveyed reliably in vocal behaviour and specific acoustic characteristics of voice, irrespective of body size and ecological niche differences between species. Cross-taxa similarities in acoustic cues of affect intensity point to phylogenetic constraints and inheritance from a common ancestor, whereas variations in vocal behaviour and affect intensity-related acoustic cues between species may be an adaptation to specific social requirements and depend on social systems. Our findings as well as a comparison with published works on acoustic communication in other vertebrate groups support the hypothesis that affect intensity in human voice originates from precursors already found deep inside the vertebrate phylogeny.

## Introduction

Language and music are multifaceted acoustic communication phenomena conveying both linguistic and paralinguistic properties, such as emotions [1]–[2]. Across cultures and languages acoustic cues in voice known to convey the quality or intensity of an emotion – the latter also called emotion or affect intensity [3] – comprise fundamental frequency (F_0_), voice intensity, duration, articulation rate or tempo (e.g. [1], [4]–[7]). These paralinguistic features in voice are part of affective prosody, i.e. patterns of stress and intonation in acoustic expressions, important not only to express what and how strongly a person feels but also to evoke or interpret these feelings in others, or to think about one's own or another's feelings to make respective decisions [8]. The fact that shared acoustic cues in affective prosody of nonverbal acoustic expressions, speech and music code for the respective quality and intensity of an emotion across human cultures [2], [8]–[16] provides support for the hypothesis that specific components of affective prosody in humans may have derived from a prehuman basis (“prehuman origin hypothesis of affective prosody” [8]). Indeed, recent findings on non-human mammals indicate that within call types used in specific contexts such as agonistic, predation, disturbance, mother-infant, group movement or foraging (birds: [17], pigs and cattle: [18], elephants: [19], bats: [20], dolphins: [21], tree shrews: [8], [22]), affect-intensity related variation in comparable acoustic features does exist. So far, to our knowledge, studies on nonhuman primates covered solely four species, ranging from nocturnal and arboreal prosimians (mouse lemurs: [23]) to diurnal and arboreal New World monkeys (e.g. squirrel monkeys: [24]) and to terrestrial Old World monkeys (e.g. macaques: [25], baboons: [26]).

To explore to which extent acoustic features coding for affect intensity are a universal trait for primates, irrespective of body size, ecological niche, and social system, we conducted the first examination of affect-related vocal behavior and acoustic features in voice in three species of forest-dwelling arboreal Old World monkeys, manipulating vocal behavior and voice by a comparable experimental and ethological approach. Following McNaugthon & Corr [27] and Altenmüller et al. [28], we postulated that the affective state of animals can be operationalized on the behavioral level by measuring the type of behavioral responses and by changes in the intensity of this response to a particular stimulus or situation.

The three studied species (i.e. De Brazza's monkey – *Cercopithecus neglectus*, Campbell's monkey – *Cercopithecus campbelli*, and red-capped mangabey – *Cercocebus torquatus*) belong to the *Cercopithecinae* sub-family and present strong eco-ethological similarities as they live in dense African primary tropical rainforests, feed essentially on fruits, defend their territory from conspecific intruders [29]–[31] and rely mainly on calls to socially communicate due to the limited visibility of their habitat [32]–[34]. The three monkey species, however, do also present divergences in body size (adult females/males weight on average 4/6, 3/5 and 6/10 kg in De Brazza's monkeys, Campbell's monkeys and red-capped mangabeys, respectively), social structure (monogamous pairs in De Brazza's monkeys, one-male multi-female groups in Campbell's monkeys and multi-male multi-female groups in red-capped mangabeys) and degree of arborealism (red-capped mangabeys spending the shortest and Campbell's monkeys the longest time up in trees, [30], [35]).

A total of five groups (Campbell's monkeys N = 1, De Brazza's monkeys N = 2 and red-capped mangabeys N = 2), maintained in similar captive conditions, were investigated comparatively. To induce a comparable change in the affective states of individuals across species, we forced a reunion of the respective social group by locking all group members together in their outside enclosure (inducing a low affect intensity condition in group members  =  low affect intensity condition). To create a change in affect intensity, the former group was first split into two subgroups for 90 min and then the two subgroups were reunited (inducing a high affect intensity condition in group members =  high affect intensity condition). The reunion in this paradigm is described to induce short-term social stress and thus a changing state of affect intensity resulting in a change of behaviors of subjects, with higher affect intensity in the pre-reunion separation condition [36]. Our expectation was that the variation in affect intensity should be reflected in vocal activity and specific acoustic features in voice. We compared call rates and voice characteristics of contact calls at the time of reunion with and without pre-separation.

We explored the following two hypotheses:

- 1 – Affect intensity is conveyed in general vocal activity across species. We expect call rates to decrease progressively after reunion with significant differences between the Low and the High affect intensity conditions.
- 2 – Affect intensity is conveyed in shared acoustic features of a structurally and contextually homologous call type across species, notably in the frequency and temporal domain [14], [20], [22], [37]. We focused on contact calls that are predominantly emitted and used during affiliative social interactions.Our prediction was that call duration and the pitch of voice will differ significantly between the Low and the High affect conditions [1] in each of the three species.

## Results

### Influence of affect intensity on the general vocal activity

**Figure 1:**
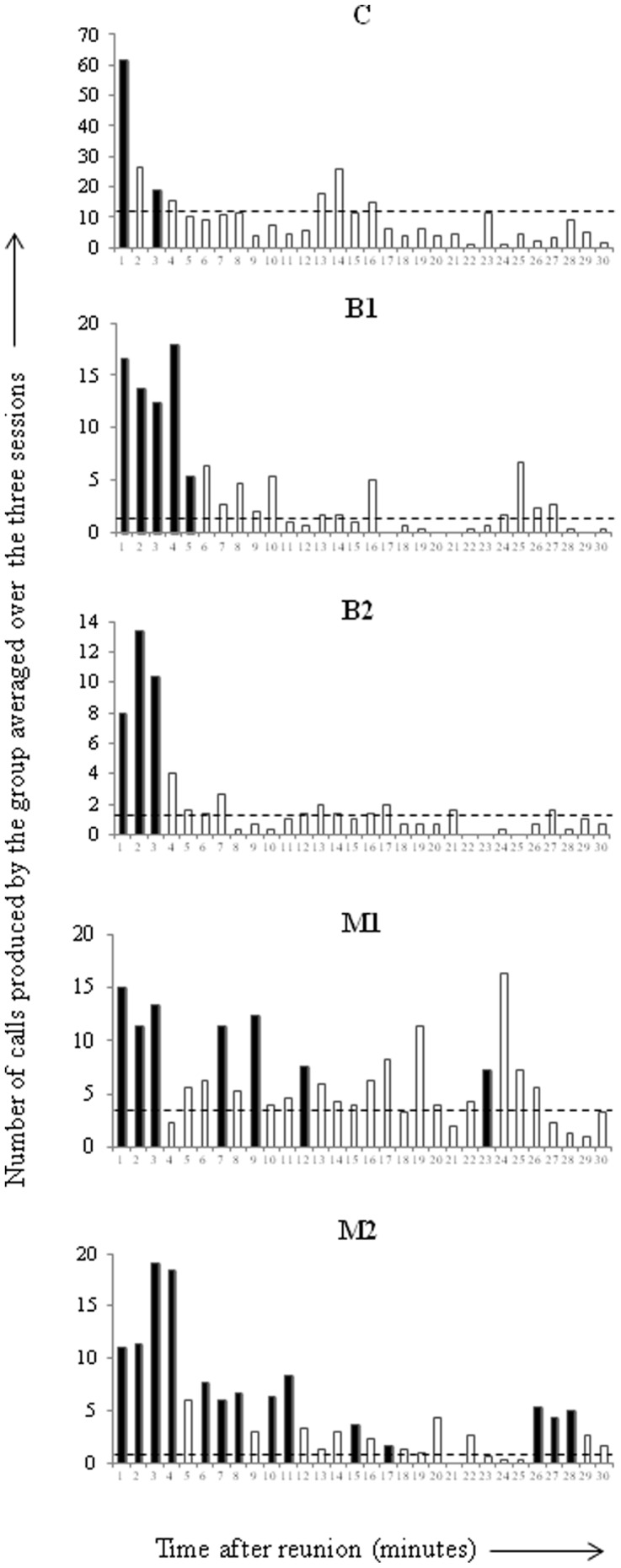
Post-reunion minute by minute temporal evolution of the mean number of calls (all call types and callers combined) emitted by each group (C, B1, B2, M1, M2) in the High affect intensity condition. Black bars represent minutes for which the number of vocalizations was significantly higher than the number of calls in the Low affect intensity condition (Mann-Whitney tests, p<0.05). Dotted lines represent mean call rates in each group in the Low affect intensity condition.

Despite strong inter-group variations, the pre-separation triggered an increase of call rates after reunion in the three studied species. Post-reunion call rates in the High affect intensity condition were significantly higher than call rates in the Low affect intensity condition in all groups (Figure 1). Moreover, the latencies to return to a Low affect intensity condition call rate in the High affect intensity condition varied between species (C = 3 mn, B1 = 5 mn, B2 = 3 mn, M1 = 23 mn, M2 = 28 mn). These findings suggest that all three species were affected by the social pre-separation and showed a progressively decreasing call rate to come back to a Low affect intensity level. Mangabeys showed a much longer latency than De Brazza's and Campbell's monkeys.

### Influence of affective state on contact call features

The acoustic structure of the contact calls emitted just after (within 5 mn) the observation started differed between the Low and the High affect intensity conditions. We found a predictable influence of the recent social separation on voice characteristics in only two species: Campbell's monkeys (Fisher's Omnibus test: ‘Low *vs* High’ Chi = 24.8 DF = 8 P<0.01) and red-capped mangabeys (‘Low *vs* High’ Chi = 26.9 DF = 8 P<0.001). No effect was found for De Brazza's monkeys (‘Low *vs* High’ Chi = 7.7 DF = 8 P = 0.47).

Nevertheless, similarly in the three species, call durations increased with increasing affect intensity (Table 1). Call durations of each species were significantly longer in the High than in the Low affect intensity condition. The same pattern was observed for frequency parameters, with higher-pitched calls emitted when the expected affect intensity increased, but only in two of the three species. Campbell's monkeys emitted higher F_max_ in the High than in the Low affect intensity condition. Red-capped mangabeys emitted higher F0_start_, F0_end_ and F_max_ in the High than in the Low affect intensity condition. De Brazza's monkeys also raised their pitch, however, not significantly.

**Table 1:** Comparison of the acoustic parameters measured on the contact calls of the three species between Low and High affect intensity conditions.

			D	F0_start_	F0_end_	F_max_
***C. campbelli***	Median ± e.s.	Low	109±9	467±27	817±143	459±32
		High	150±7	528±34	1094±158	552±44
	Wilcoxon p/T	Low *vs* High (N = 11)	**0.003/0**	0.214/12	0.183/9	**0.035/2**
***C. neglectus***	Median ± e.s.	Low	146±16	1106±100	1106±100	2955±228
		High	238±21	1138±72	1138±72	3077±236
	Wilcoxon p/T	Low *vs* High (N = 5)	**0.043/0**	0.855/5	0.855/5	0.686/6
***C. torquatus***	Median ± e.s.	Low	62±5	438±22	438±22	438±22
		High	113±6	527±24	527±24	527±24
	Wilcoxon p/T	Low *vs* High (N = 7)	**0.018/0**	**0.043/0**	**0.043/0**	**0.043/0**

First row: median ± e.s. value of the acoustic parameters (see definitions in the method section). Second row: results of the Wilcoxon tests (p/T values). Bold numbers indicates P<0.05 values.

The number of individuals (N) included in the Wilcoxon analysis is given between brackets.

## Discussion

The three studied species reacted in a very similar way to our experimental social separations. Call rates in the High affect intensity condition decreased progressively after reunion to reach a Low affect intensity condition. Differences between mangabeys and guenon species were observed with regards to the latency needed to come back to Low affect intensity condition. Affect intensity, induced by the presence/absence of the pre-reunion separation, was conveyed in shared acoustic features, notably longer call durations in three of our species and higher-pitched frequencies in two species only.

**Figure 2:**
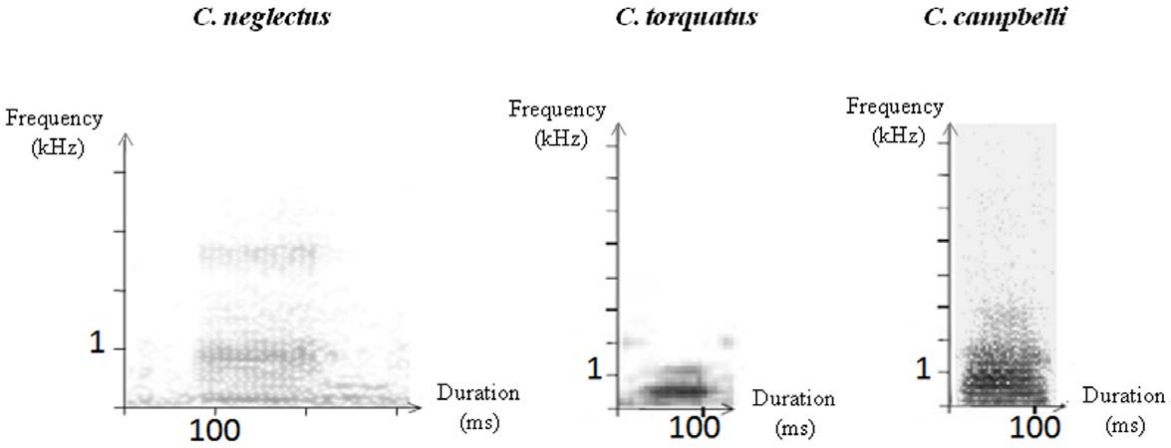
Sonograms of the contact calls in the three studied species.

Affect intensity appeared to be conveyed in global vocal activity with increased call rates at the reunion time in all species studied. In the wild, subgroup encounters are often associated with increased call rates, notably in species with fission-fusion social systems (killer whales: [38], dolphins: [39], elephants: [40]). The same increase of call rates linked to an increase in affect intensity is, however, also observed across a broad range of animal taxa from frogs to primates (e.g. strawberry poison-dart frogs [Pröhl, pers. Communication], birds [17], [41], [42], pigs and other mammals: [43]) suggesting a deep phylogenetic origin in the common ancestor of all voice-producing vertebrates. Hence, vocalisations are said to be relevant to measure farm animal (poultry, cattle, pig) welfare as calls can be elicited by the injection of drugs that stimulate neuronal circuitries involved in mood and emotions (for a review see [18]). Interestingly, the chick arousal state (induced experimentally by a progressive social isolation) modifies call rates, especially calls with high energy, such as distress calls [44]. Reunion after experimental separation in captive Tonkean macaques was associated to polyadic intense affiliative gestural and vocal expressions [36]. In our study, vocalisations seemed to be the only communicative signals impacted. Our species are arboreal forest-dwelling monkeys living in dense vegetation and they rely mainly on sounds to communicate with much rarer gestures than other old world monkeys like more terrestrial adapted monkeys such as macaques or baboons [45]. Hence, the high rates of calls after reunion in our species may be explained by three non exclusive hypotheses. First, calling may be an external manifestation of individually experienced stress due to the recent separation. Thus, neurological studies evidenced that the production of vocalisations in monkeys is mainly uncontrolled and processed in subcortical areas associated with emotion (e.g. limbic system) [46]. Second, it could reflect a massive and collective emotional discharge of vocalizations linked to joyful feelings at the time of reunion spreading within the group through a phenomenon of social contagion or social facilitation leading to a rapid increase of call rates at the group level [47]. Third, another possibility is that vocalizations will be intentionally uttered with some bonded individuals exchanging calls with one another in order to rapidly re-establish social cohesion and advertise social affinities [48]–[49]. We frequently see an increase of affiliative behaviours after reunion in animals (elephants: [50], spotted hyenas: [51], chimpanzees: [52]–[53], but also an increase of agonistic behaviours (chimpanzees: [54], spider monkeys: [55]). Support for a strategic use of calls comes from a recent study which suggests a monkey homolog of Broca's area (ventral premotor cortex) for voluntary vocalizations [56].

Differences between species were observed in the latency to return to Low affect intensity condition. The latency was much higher in mangabeys than in the two guenon species. Social factors may be good predictors for the observed changes. Guenons are said to have a social organization based on a « monitor - adjust » system characterized by a limited number of physical interactions and large inter-individual distances while mangabeys have a social organization more like the ones of macaques and baboons with small inter-individual distances and more frequent interactions [57]–[58]. It is thus possible that being physically apart is more disturbing for mangabeys than guenons.

Separation induced a strong global change in the voice of callers in two species (Campbell's monkeys and red-capped mangabeys) and slighter changes in De Brazza's monkeys. Body size and ecological niche do not explain the interspecific difference as De Brazza's monkeys present profiles intermediate to Campbell's monkeys and red-capped mangabeys. Again, social factors may better explain the difference. De Brazza's monkeys are characterized by a much less complex social life (uni-matriline) than the two other species. A social disturbance, as used in this study to change individuals' affect, may not be the most appropriate method for such species. When looking at each acoustic parameter separately, we found that affect intensity was conveyed in comparable acoustic features of voice. In all species call duration was longer when the affect intensity was the highest. This phenomenon is also observed in humans [1] and a large range of mammals: macaques [59], baboons [26], bats [20], tree shrews [22], elephants [60] and dolphins [21]. In Campbell's monkeys and red-capped mangabeys, pitch frequencies were also higher when the affect intensity was higher (this frequency switch was also found in De Brazza's monkeys but was not significant). Similar shifts in frequency encoding emotions were found in humans [9], baboons [26], squirrel monkeys [24], tree shrews [22], bats [20] and dolphins [26]. Since energy distribution is related to the form of the vocal tract [61], and since call duration is dependent on the amount of air available, we can assume that the internal affect modify the monkey's physiology, posture and breathing activity, in a way similar to humans. The shift in fundamental frequency may be related to changes in subglottal air pressure and general muscle tone of the vocal folds, both effects of sympathetic arousal [37]. Although, non-mammalian studies are still limited on this topic, there is no doubt that the relation between the arousal state of the caller and its voice characteristics is not limited to mammalian species. Despite differences between the vocal apparatus of mammals and birds, which theoretically may lead to a different acoustic impact of the arousal state, interestingly, Perez et al. [62] have also observed an increase of call duration and pitch with the level of arousal in zebra finches. The increase of pitch was also found in aggressive contexts in Swamp sparrows [42]. At last, the signaler's perception of urgency of a danger is coded in both birds (e.g. fowl [63]) and mammals (e.g. suricate [64], monkeys [65]) alarm call acoustic structures.

In sum, the duration parameter appeared predominant in the process of affect encoding here, whatever the species body size and socio-ecological life. We acknowledge that the conclusions of this work are based on relatively small sample sizes for the three investigated species. More comparative work is now needed to understand to which extent these physiological effects linked to the variation of an internal affect state are universally spread in the primate lineage as well as in voice-producing vertebrates at all, and to be able to trace evolutionary pathways of the origin of the voice of emotion in human speech.

## Materials and Methods

### Study groups

We conducted our study on five captive social groups: one group of Campbell's monkeys (‘C’ composed of 8 adult females, 2 subadult females, 1 subadult male, 1 juvenile male and 1 juvenile female), two groups of De Brazza's monkeys (‘B1’ composed of 2 adult females, 1 subadult male and 1 juvenile male – ‘B2’ composed of 1 adult male, 1 adult female and 2 juvenile females) and two groups of red-capped mangabeys (‘M1’ composed of 1 adult male, 3 adult females, 1 subadult male, 1 subadult female, 3 juvenile females and 2 juvenile males – ‘M2’ composed of 3 adult females and 2 juvenile males). All individuals were captive-born. The five groups were housed in the primate centre of Rennes 1 University in outdoor (ranging from 10 to 300 m^2^×4 m high) – indoor (ranging from 10 to 15 m^2^×3 m high) enclosures enriched with branches and cords for climbing and straw litter inside. Monkeys were fed with fruits and vegetables in the morning and chow in the afternoon after the experiments. Water was available *ad libitum*. Animal care and research protocols used in this work complied with the current French laws governing animal research and were approved by the ‘Direction Departementale des Services Vétérinaires’ ethic committee (permit number #04672). Animal welfare was strictly respected given that our experiment was totally non-invasive involving only observations without any animal manipulation and only short-term spatial restriction in their own familiar enclosure with no individual being isolated.

### Experimental procedure

Experiments were conducted in April and May 2011 under two conditions. We recorded during 30 minutes after a forced reunion the vocal behaviour of all group members. In one condition, all group members were free to move in their enclosure prior to the reunion (Low affect intensity condition). In the other condition, the group was split into two sub-groups for 90 minutes before reunion (High affect intensity condition). Each experimental condition was repeated three times for each group and the order of sessions was randomized. To be comparable, observations with or without pre-separation, were conducted once a day per group with the reunion occurring at fixed hours of the day (C: 16 h30, B1 and B2: 15 h, M1 and M2: 11 h) and with at least one-day break between two consecutive sessions.

#### Separation and reunion procedure

In the High affect intensity condition, each group was separated in two same-sized (+/− 1) sub-groups (one left indoor and the other one left outdoor) 90 minutes before the reunion time. Subgroups could hear and see each other through windows but could not physically interact with one another. Sub-group compositions were randomized and changed at each session to avoid possible biases due to individual preferences. At reunion time, the inside sub-group was moved outside through a trap door by the caretaker (AR) and the whole group was locked outside for the subsequent observations. The Low affect intensity condition consisted in moving any animals that were inside to the outside and closing the trap door without forced pre-separation.

#### Observation procedure

All calls produced in the two conditions were recorded under an all-occurrence sampling regime [66] during 30 minutes after the trap door was closed. The experimenter (KR) was still and stood always at the same location near the enclosure in separation and control conditions. Recordings were done with a digital stereo recorder (Marantz® PMD660, sample rate  = 44100 Hz, resolution  = 16Bits) connected to one directional microphone (Sennheiser® K6/ME66, for the animal calls) and to one tie microphone (Sony® ECM-T6, for the experimenter comments). The experimenter identified callers each time it was possible (i.e. caller clearly visible and no call overlap).

### Data analysis

#### Influence of affect intensity on the general vocal activity

We recorded the precise moment of emission of each call uttered in the studied group by listening to each recording using Audacity® (V 1.2.6) software (time resolution: 10^−2^ seconds). In order to analyze the effect of the experimental condition on the temporal evolution (minute by minute) of call rates (all call types and all callers combined) in the five studied groups, we conducted Mann-Whitney U tests for each group. For each group, regardless of caller identity, we compared the number of calls of each single minute in the three sessions of the High affect condition with the call rates per minute recorded in each of the three sessions of the Low affect condition. We expected to find higher rates in the High than in the Low affect condition [36].

#### Influence of affect intensity on voice: contact call features

We conducted acoustic measurements on the contact calls of the three species (see for definition of the contact call types in the three species: [32]–[34]; Figure 2). Sonograms were drawn with ANA software [67] by conducting a 256-point Fast Fourier Transformation (FFT) with a time resolution of 1ms and a frequency resolution of 86 Hz. As their contact calls are optionally composed of several sound units (De Brazza's monkey [34] and Campbell's monkey [32]: low-pitched grunt followed by an optionally high-pitched component, mangabey [33]: low-pitched grunt repeated several times in a row), we measured only the first grunt-like unit in order to standardize comparisons. We conducted acoustic measurements on all calls emitted by an individually identified caller, within the first 5 minutes of the observation period (for both Low and High affect conditions), when the impact of the forced reunion was supposed to be the strongest (Number of calls measured: n_Campbell_ = 208, n_Brazza_ = 282, n_mangabey_ = 142). Acoustic parameters were: duration [D, ms], fundamental frequency [F0_start_/F0_end_, Hz, respectively measured at the beginning and at the end of the call], dominant frequency [F_max_, Hz, frequency presenting the highest intensity].

To examine to what extent affect intensity will influence contact calls in the respective species, we compared, at the individual level for each species, acoustic parameters between the Low and the High affect intensity conditions using a non-parametric test for dependent data, the Wilcoxon Signed Rank test [68]. To bring about a global decision on the null hypothesis (the recent separation has no predictable inﬂuence on the acoustic structure of the contact calls), we used the Fisher's Omnibus Test [69], as has been done in similar studies (e.g. [37]). This test uses the multiple P values produced by the Wilcoxon Signed Rank tests to create an overall P value. This overall P value resulted in a overall acceptance or refusal of the null hypothesis and hence put aside α-adjustments for each variable which would have been necessary when testing the same null hypothesis several times.
